# Motivational Drivers of Temporal Dynamics in Postretirement Work

**DOI:** 10.1093/geronb/gbac130

**Published:** 2022-09-08

**Authors:** Isabelle Hansson, Kène Henkens, Hanna van Solinge

**Affiliations:** Department of Psychology, University of Gothenburg, Gothenburg, Sweden; Centre for Ageing and Health (AgeCap), University of Gothenburg, Gothenburg, Sweden; Netherlands Interdisciplinary Demographic Institute (NIDI-KNAW), The Hague, The Netherlands; Netherlands Interdisciplinary Demographic Institute (NIDI-KNAW), The Hague, The Netherlands; Department of Health Sciences, University Medical Center Groningen, Groningen, The Netherlands; Department of Sociology, University of Amsterdam, Amsterdam, The Netherlands; Netherlands Interdisciplinary Demographic Institute (NIDI-KNAW), The Hague, The Netherlands; Department of Health Sciences, University Medical Center Groningen, Groningen, The Netherlands

**Keywords:** Aging workforce, Bridge employment, Retirement, Work motivation

## Abstract

**Objectives:**

Many retirees continue to work in retirement, but the temporal dynamics of this process are not well understood. This article examined the extent to which retirees increase, decrease, and exit their work engagement over time. We hypothesized that different motives for postretirement work—financial, social, personal, and organizational—have differential affects on changes in work extent.

**Methods:**

We analyzed 7 waves of the HEalth, Aging and Retirement Transitions in Sweden study (*n* = 3,123). Postretirement work was defined as working for pay while receiving pension benefits. Changes in work extent were estimated with multistate models and examined in relation to the 4 motives.

**Results:**

Results showed a gradual decrease in work extent following retirement. Financial motives increased the likelihood to take up more work and decreased the likelihood to reduce work hours. Social motives increased the likelihood to reduce and exit work, while personal motives decreased the likelihood for those same pathways. Organizational (demand-driven) motives increased the likelihood to stop working.

**Discussion:**

Our findings suggest that financial motives constitute an important driver for taking up more work in retirement, while motives related to the personal meaning of work explain why retirees maintain their level of engagement over time. The social function of work, on the other hand, may be gradually replaced by social activities outside of work, resulting in a gradual disengagement from work. Finally, demand-driven motives appear insufficient to remain in the labor force, highlighting the need to acknowledge the diversity of motives for continuing to work.

Rapid demographic change combined with more flexible pension arrangements have led to a diversification of the retirement landscape in most developed countries. Slowly but surely, the traditional view of retirement as a one-step permanent career exit has been replaced by a more nuanced picture in which the diversity of pathways are acknowledged (Ekerdt, 2010; Shultz & Wang, 2011). Retirement in the 21st century is viewed as a complex process unfolding over time, and the timing and design of this process may vary considerably between individuals (Henkens & van Solinge, 2021). The boundaries between work and retirement have been blurred as more and more retirees continue to work in retirement (Cahill et al., 2015; Giandrea et al., 2009). In this article, we focus on those who continue to work after they have started to take out old-age pension, and we use the term postretirement work rather than bridge employment because of the varying definitions of this concept in the literature (Beehr & Bennet, 2015; Cahill et al., 2018). A growing body of research demonstrates heterogeneity in the prevalence (across countries and subpopulations) and nature (e.g., same vs different occupations) of different types of postretirement employment (Alcover, 2017; Beehr & Bennet, 2015). However, our understanding of the temporal dynamics, that is, the extent to which retirees increase or decrease their work engagement over time, is still limited.

A thorough understanding of the retirement process requires attention to when, how, and why retirees continue to work in retirement. Knowledge about the temporal dynamics of this process is important to gain insights into the driving mechanisms behind postretirement work, but also for developing strategies to meet the demands of a rapidly aging population. In this article, we argue that to understand this dynamic, it is important to incorporate the motivational drivers for taking up work in retirement. We specifically aim to investigate: (a) the extent to which retirees increase, decrease, and exit postretirement work, and (b) if their motives for continuing working account for individual differences in this process. To answer these research questions, we utilize data from a Swedish longitudinal survey on retirement.

## Temporal Dynamics of Postretirement Work

Postretirement employment can take many forms (e.g., part-time, full-time, or self-employment), and the same person may engage in different types of jobs (e.g., same vs different occupation) over an extended period of time (Alcover et al., 2014; Beehr & Bennet, 2015). The wide range of possible employment arrangements pose challenges not only in defining and measuring the prevalence of postretirement work, but also in comparing the prevalence across populations and national contexts. Research, however, suggests an emerging trend where more and more people engage in paid work after retirement (Cahill et al., 2015; Henkens & van Solinge, 2021), and Sweden is no exception (Nilsson & Ferm, 2021). A growing body of literature has documented the prevalence of different types of postretirement employment (e.g., Cahill et al., 2018) and the characteristics of those who engage in them (e.g., Dingemans et al., 2017). The implicit assumption in this research is that it facilitates a gradual disengagement from work and serves as an intermediate step toward complete withdrawal from the labor force. As a result, studies typically focus on identifying the determinants and outcomes of postretirement employment rather than examining how the level of work engagement changes over time. The extent to which retirees increase or decrease their work hours in retirement is therefore largely overlooked.

Most of what we know about postretirement work is based on either cross-sectional studies comparing working and nonworking retirees (e.g., Dingemans et al., 2017; Fasbender et al., 2016) or prospective studies on workers transitioning to partial versus full retirement (e.g., Cahill et al., 2018; Dingemans et al., 2017; Wang et al., 2008; Zhan et al., 2015). These studies are informative for understanding individual differences in postretirement employment participation, showing, for example, that men and individuals with higher education and better health are overrepresented among those who continue to work in retirement (see Beehr & Bennet, 2015 for an overview), but they provide limited insights into the longitudinal process in which workers gradually withdraw from the labor force. A thorough understanding of the nature of postretirement employment as well as the characteristics of those who engage in them therefore requires attention to how the level of engagement changes over time. By focusing on the temporal dynamics rather than snapshot images of individual statuses or transitions, we gain a more detailed understanding of when, how, and why retirees continue to work in retirement.

In one of the few studies incorporating a dynamic perspective, Calvo et al. (2018) analyzed retirement sequences of older Americans and identified groups of individuals with similar retirement patterns. This study captures the diversity of pathways to retirement and provides insights into how individuals move in and out of work before fully withdrawing from the labor force. However, it is limited in that it only differentiates between partial and full retirement and therefore does not inform us regarding changes in postretirement work extent. In a similar study, Tang and Burr (2015) examined dynamic pathways between different types of work and retirement statuses, but they also focused on transitions between partial and full retirement rather than the degree of work participation. Thus, we still lack in our understanding of the extent to which retirees change their level of work engagement over time.

In this article, we argue that a more fine-grained analysis of temporal changes in postretirement work is necessary to gain a better understanding of the retirement process and the factors contributing to prolonged labor force participation. For this purpose, we investigate annual changes in postretirement work extent in a sample of older adults from the HEalth, Aging, and Retirement Transitions in Sweden (HEARTS) survey (Lindwall et al., 2017). Sweden has a flexible pension system with eligibility for a public pension from the age of 61 and the legal right to remain in employment until the age of 67 (at the time of data collection). The public pension is income-based (with special social security benefits for individuals with low or no income) and is supplemented by occupational pension (eligibility age varies depending on the collective agreement) and private pension savings. The average net pension replacement rate is 55%, which is lower than the Organisation for Economic Co-operation and Development (OECD) average (63%; OECD, 2017), but the risk of old age poverty or social exclusion is relatively low (15%, EU average 19%; European Commission, 2021). While it is possible to combine work and public pension benefits at varying rates, the vast majority take out 100% of their pension, and most of those with continued employment work part-time (Nilsson & Ferm, 2021). The high employment rates among older adults (OECD, 2017) and the large proportion of individuals who engage in postretirement work (Dingemans et al., 2017) make Sweden an interesting country of reference to better understand the factors involved in shaping postretirement employment decisions and behaviors. The HEARTS study contains seven annual measurement waves and detailed information on the level of work engagement, which offer valuable opportunities to examine drivers of change in postretirement work extent. In this article, we argue that the motives for continuing working play a significant role in understanding why retirees change (or exit) their work engagement over time. Therefore, in the next section, we address how different types of motivations may shape postretirement work decisions and behaviors.

## Reasons for Working in Retirement

Central questions for understanding the motivational drivers behind postretirement work concern the meaning of work, the different functions it serves, and the degree to which it helps to fulfill important needs in retirement. Aspects of role loss in the retirement transition (Ashforth, 2000) and the need to maintain continuity in internal and external structures (Atchley, 1989) are key mechanisms in this regard (e.g., Wang et al., 2008). In the process of adapting to life in retirement, retirees may seek continued employment to avoid the abrupt work role loss and the break of continuity of life patterns (Zhan & Wang, 2015). However, depending on the specific aspects of work one expects to miss, the motives for engaging in postretirement work may differ across individuals (Dendiger et al., 2005; Loi & Shultz, 2007; Mor-Barak, 1995). In this article, we argue that different motives also vary in their association with changes in work extent. As a theoretical foundation for this assumption, we address the different functions of work as suggested by Beehr and Bennet (2015).

Beehr and Bennet (2015) discuss the manifest (financial) and latent (time structure, social contact, collective purpose, activity, and identity or status) functions of work (as proposed by Jahoda, 1997), and how they may influence postretirement work engagement. They argue that the prospects of missing one or more of these functions of work motivate retirees to continue working. As long as there is a need for these functions of work, and as long as the needs are met by the work, individuals are expected to stay in postretirement employment. As time passes and people gradually adjust to retirement, needs may become more or less important or be met elsewhere (e.g., through nonwork activities). As a result, retirees may choose to increase, decrease, or exit their work engagement. Based on this assumption, we expect motives to differ depending on the needs of the individual, but also in how they influence changes in work participation. In this study, we focus on motives related to financial, social, personal, and organizational needs, and examine how they relate to changes in work extent.

The financial function of work is perhaps the most obvious in discussions regarding motives for postretirement work. As most retirees face a significant drop in income following retirement (OECD, 2017), we expect economic resources to have a direct influence on work and retirement decisions. Research confirms this assumption and shows that financial motives are important for understanding why retirees continue to work in retirement, although it has been shown to be less prevalent than social and personal motives (Dingemans & Henkens, 2014; Hansson et al., 2020; Mazumdar et al., 2020; Platts et al., 2021). The financial meaning of work is suggested to be a stronger predictor of postretirement employment among retirees with poor economic status (Fasbender et al., 2016), and financial motives are related to a preference to work more hours in retirement (Hess et al., 2021). Research on older workers further demonstrates that many part-time jobs are involuntary, that older workers often want to work more, and that financial strain is one of the main motivators to increase work hours (Silver et al., 2019; Starace et al., 2015; Van Horn & Heidkamp, 2019). Because the financial function of work is difficult to find elsewhere, we hypothesize that retirees who report financial motives for continuing to work are more likely to increase their work engagement over time (Hypothesis 1a). We further expect that retirees who work for financial reasons have a continuous need for occupational income due to restricted opportunities to meet this need elsewhere, which would make them less likely to decrease (Hypothesis 1b) and exit (Hypothesis 1c) work.

Motives related to the social function of work highlight the need to maintain work-related social ties in retirement. Research has shown that retirees who value the social meaning of work, as well as those anticipating social loss in retirement, are more likely to plan for and take up postretirement work (Fasbender et al., 2014, 2016; van Solinge et al., 2021). However, working for social reasons does not necessarily translate into a desire to work more (Hess et al., 2021; Mazumdar et al., 2020; Platts et al., 2021). Research suggests that work-based relationships often are integrated into personal networks, and that work-related personal ties are maintained after retirement (Cozijnsen et al., 2010). The social function of work may therefore be less dependent on the degree of work engagement but rather facilitate a gradual shift from work to nonwork-related social engagements. As retirement brings more time to build on social relations outside the workplace, we expect that retirees will gradually seek to replace social contacts at work with social activities outside of work. We specifically hypothesize that retirees who report social motives for continuing working are less likely to increase (Hypothesis 2a) and more likely to decrease (Hypothesis 2b) their work engagement over time. Based on the anticipation of replacement mechanisms in the need of work to maintain social ties, we further expect that those who work for social reasons are more likely to exit from the labor force (Hypothesis 2c).

Motives related to the personal meaning of work highlight the need to mitigate experiences of role loss and to maintain one’s job identity in retirement. Research shows that personal motives such as finding a sense of purpose, meaning, or enjoyment from work constitute the most important reason to engage in postretirement work (Dingemans & Henkens, 2014; Fasbender et al., 2016; Hansson et al., 2020; Mazumdar et al., 2020; Platts et al., 2021). While personal motivations may be important for obtaining a preferred level of work engagement, we do not expect it to play a significant role in understanding why retirees increase their work extent over time. Instead, based on the assumption that functions related to the personal meaning of work are difficult to find and replace elsewhere, we hypothesize that those who work for personal reasons opt for continuity and therefore are less likely to decrease (Hypothesis 3a) and exit (Hypothesis 3b) their work.

Finally, as the opportunity for postretirement work may be conditioned on employer demand, we additionally examine motives related to organizational needs. While demand-driven motives may generate personal motivations to contribute and to play a significant role in the organization, they are by definition conditioned on the needs of the employer. Even though research shows that employees usually take the initiative in prolonging employment (Tunney & Oude Mulders, 2021), their ability to continue working ultimately depends on the employer’s willingness to recruit or retain them (Vickerstaff, 2006). Studies on employers’ attitudes toward older workers suggest that managers often are disinclined to rehire retirees, but also that their willingness depends on the organizational context and business cycle (Karpinska et al., 2011; Oude Mulders et al., 2014). Based on the assumption that employer demands are temporary and conditioned on external factors, we hypothesize that retirees who report motives related to organizational demands are more likely to decrease (Hypothesis 4a) and exit (Hypothesis 4b) the labor force. Because the need of the employer may vary independently of employee motivations, we do not expect organizational motives to predict an increase in work extent over time.

## Method

### Participants

The present study is based on seven waves of annual measurement data from the HEARTS study (Lindwall et al., 2017), a longitudinal population-based survey of older adults in Sweden born between 1949 and 1955 (age 60–66 at baseline). The first survey was distributed in 2015 (*N* = 5,913; response rate 39.4%), with annual follow-ups conducted in 2016 (*N* = 4,651, 78.7% of baseline sample), 2017 (*N* = 4,320, 73.1% of baseline sample), 2018 (*N* = 4,033, 68.2% of baseline sample), 2019 (*N* = 3,935, 66.6% of baseline sample), 2020 (*N* = 3,914, 66.2% of baseline sample), and 2021 (*N* = 3,660, 61.9% of baseline sample). Details on the study design, measurement domains, and previous publications can be found at https://osf.io/wcbxu/.

For this study, we included individuals who were retired at baseline or retired during the study period (*n* = 4,729). Retirement was defined as receipt of old-age pension and indicated (in the survey) with the date of first pension withdrawal. Participants were excluded if they: (a) retired before age 61 (lowest public pension age in Sweden at the time of data collection, *n* = 323), (b) did not work (due to unemployment or disability) before retirement (*n* = 605), (c) only completed the baseline assessment (*n* = 563), and (d) only reported their work status once (i.e., information on work status missing at follow-up; *n* = 115). The final sample consisted of 3,123 individuals who provided a total of 13,742 observations. The participants had a mean age of 67.44 years across observations (standard deviation [*SD*] = 2.18, range 61–72), the average retirement age was 64.14 (*SD* = 1.50, range 61–71), and they had spent an average of 3.12 years in retirement (*SD* = 2.07, range 0–11).

### Measures

#### Postretirement work

Postretirement work was measured in each wave with the question, “To what extent do you currently work?” We differentiated between (a) “Working full-time,” (b) “Working a few days a week,” (c) “Working once a week,” (d) “Working once or twice a month,” and (e) “Not working, full-time retiree.”

#### Reasons for working in retirement

Reasons for working in retirement were measured in wave two to six. Working retirees were asked about their reasons for continuing working by indicating the extent to which different statements with possible reasons applied to them. The four motives—financial (“My financial situation demands it [cannot afford to stop]”), social (“The social contact with others is important to me”), personal (“I want to continue to do something meaningful”), and organizational (“I was needed at my old job”)—were measured with one item each. Response alternatives ranged from completely false (1) to completely true (5).

#### Demographic characteristics

As opportunities for, and engagement in, postretirement work may be constrained by factors operating at multiple levels (e.g., Carr et al., 2021; Maestas, 2010), we included years spent in retirement, retirement age, gender, relationship status, spouse’s work status, total pension income, involuntary retirement, occupational status, caregiving, depressive symptoms, and functional limitations as covariates. The number of years in retirement was calculated based on the reported retirement date and date of survey response, and the retirement age was calculated from retirement date and date of birth. Gender (male = 0, female = 1) was measured at baseline, and marital status (single/divorced/widowed = 0, married/partnered = 1), spouse’s work status (not working = 0, working = 1), caregiving (helping parents/in-laws/children/grandchildren every week or more often; no = 0, yes = 1), depressive symptoms (11 items; Radloff, 1977; range 0–33), and functional health limitations (no = 0, yes, to some extent = 1, yes, to a large extent = 2) were measured in all seven waves. Involuntary retirement (completely voluntary = 1, completely involuntary = 5) was taken from the first measurement occasion in retirement. Total pension income in the first full year after retirement (transformed, 1 = 100,000 SEK) and occupational status (white-collar [ISCO-08 group 1–4] = 0, blue-collar [ISCO-08 group 5–9] = 1) was drawn from the registry database (longitudinal integrated database for health insurance and labor market studies).

### Statistical Analyses

Changes in postretirement work engagement were investigated with multistate models using the msm package (Jackson, 2011) in R (R Core Team, 2021). Multistate modeling is a flexible method for analyzing the process in which an individual moves through a series of states in continuous time (Andersen & Perme, 2008; Commenges, 1999; van den Hout, 2016). The model is based on the Markov assumption (Kalbfelisch & Lawless, 1985), and the information that is used to estimate the model parameters is based on pairs of consecutive observations. The number of observed individual transitions between each pair of states consequently restricts the number of parameters that can be estimated (Jackson, 2011). As a first step, we therefore examined the frequency of transitions between the five work states: (a) “Working full-time,” (b) “Working a few days a week,” (c) “Working once a week,” (d) “Working once or twice a month,” and (e) “Not working, full-time retiree.” Because of a relatively small number of transitions between specific work categories (Table 1), we decided to constrain the covariate effects to be equal for all increase (a), decrease (b), and exit (c) transitions (i.e., reducing the number of estimated parameters). To further facilitate parameter estimation, we analyzed the three pathways (increase, decrease, or exit) in separate models. For each of the three models, we selected a subsample of participants who either transitioned or remained in the original work state across two measurement points (i.e., only participants who could potentially transition between these states were included). Reasons for working (financial, social, personal, and organizational) were included as predictors of change in work extent in the following year (i.e., increase, decrease, or exit is predicted from the motive of the previous year). In the next step, we included the demographic characteristics as covariates.

**Table 1.** Frequency of Transitions Between Different Types of Postretirement Work Status

	To				
From	Not working	Working once or twice a month	Working once a week	Working a few days a week	Working full-time
Not working	6,967	120	52	21	1
Working once or twice a month	191^a^	554	120^b^	35^b^	6^b^
Working once a week	83^a^	202^c^	331	119^b^	8^b^
Working a few days a week	99^a^	118^c^	225^c^	782	51^b^
Working full-time	23^a^	24^c^	28^c^	157^c^	302

*Notes*: *n* = 3,123, *obs*. = 13,742.

^a^Exit transitions (total = 396).

^b^Increase transitions (total = 339).

^c^Decrease transitions (total = 754).

## Results

**Figure 1.**
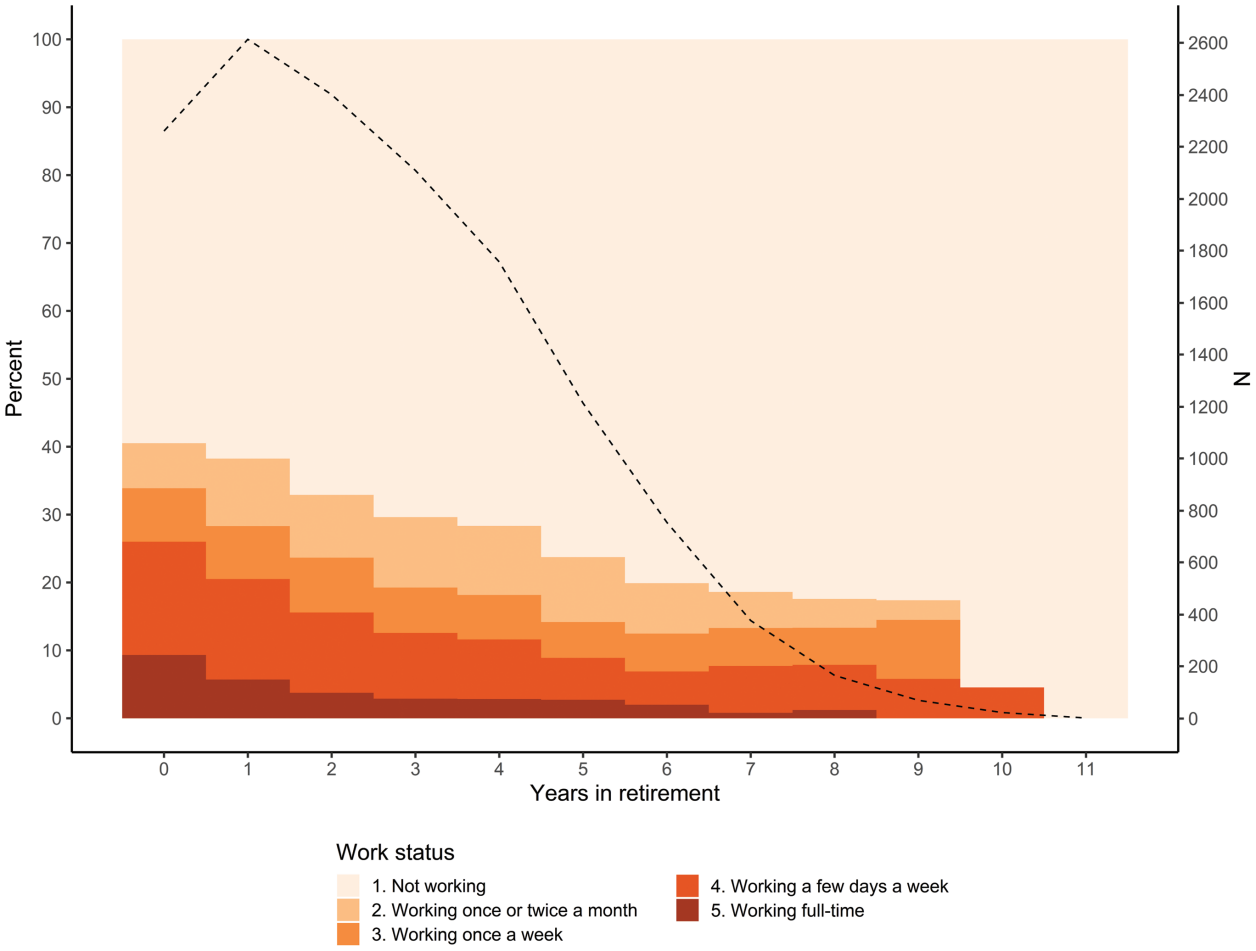
Proportion of working retirees in relation to the retirement event (date of pension withdrawal).*Notes*: The dotted line (second *y*-axis) shows the number of individual observations for each year in retirement.

On average, across measurement waves, about one third of the retirees were working (Table 2), but the proportion and degree of work engagement decreased with more time spent in retirement (Figure 1). In the first year following retirement, 23.0% of the working retirees (i.e., categories 2–5 in the figure) were working full-time, and 5 years later, the corresponding number was 11.5%. At the same time, the proportion of individuals working once or twice a month increased from 16.4% to 40.3% (of those who were working) from the first to fifth year of retirement. The transition frequencies in Table 1 additionally show that retirees were more likely to move to a closer work category (e.g., from a few days a week to once a week) than to take bigger steps (e.g., from a few days a week to full-time) in work involvement. About one in five (21.3%) reported financial motives (i.e., response alternative 4 and 5 combined), 67.7% social, 72.7% personal, and 44.7% organizational (Table 3). Financial and organizational motives were relatively unrelated to the other motives, while a substantial overlap was found between social and personal motives (*r* = 0.51–0.61 across waves; Supplementary Table S1). Descriptive statistics (mean, *SDs*, and correlations) of all study variables can be found in Supplementary Table S2.

**Table 2.** Frequency Distribution of Postretirement Work Status at Each Wave of Assessment

	T1		T2		T3		T4		T5		T6		T7	
	*n*	%	*n*	%	*n*	%	*n*	%	*n*	%	*n*	%	*n*	%
Not working	724	69.15	957	65.19	1,219	65.15	1,406	66.70	1,575	66.99	1,800	69.93	1,682	72.41
Working	323	30.85	511	34.81	652	34.85	702	33.30	776	33.01	774	30.07	641	27.59
Once or twice a month	57	17.65	126	23.66	164	25.15	180	25.64	236	30.41	260	33.59	208	32.45
Once a week	74	22.91	97	18.98	136	20.86	153	21.79	164	21.13	189	24.42	161	25.12
A few days a week	121	37.46	193	37.77	249	38.19	264	37.61	275	35.44	239	30.88	219	34.17
Full-time	71	21.98	95	18.59	103	15.80	105	14.96	101	13.02	86	11.11	53	8.27

**Table 3.** Mean, Standard Deviation, and Frequency Distribution of Reasons for Working in Retirement

Motive	Item	*obs*.	*M*	*SD*	1 Completely false	2	3	4	5 Completely true
Financial	My financial situation demands it (cannot afford to stop)	2,953	2.21	1.42	48.09%	15.54%	15.07%	9.96%	11.34%
Social	The social contact with others is important to me	3,029	3.86	1.05	3.43%	7.10%	21.72%	35.72%	32.02%
Personal	I want to continue to do something meaningful	3,021	3.97	1.06	3.44%	6.49%	17.41%	35.15%	37.50%
Organizational	I was needed at my old job	2,924	2.91	1.68	37.76%	5.44%	12.14%	16.93%	27.74%

*Note*: Data aggregated across measurement points (T2–T6). *SD* = standard deviation.

The left column in Table 4 shows the estimated associations for increase in work extent. Increasing the engagement in postretirement work was more likely among those who reported working for financial reasons (supporting H1a). No associations were found with the extent to which working retirees reported social, personal, and organizational motives. Retirees with a partner and those reporting involuntary retirement were more likely to take up more work, while functional limitations decreased the likelihood to increase work. Retirement age, number of years spent in retirement, gender, spouse’s work status, pension income, occupational status, caregiving, and depressive symptoms were unrelated to increase in work extent. The inclusion of demographic variables in Model 2 additionally increased the confidence interval for the financial motive (resulting in a nonsignificant association), but the effect size was only marginally reduced.

**Table 4.** Hazard Ratio and 95% Confidence Intervals for the Effects of Reasons for Working in Retirement on Change (Increase, Decrease, or Exit) in Postretirement Work Status

	Increase^a^	Decrease^b^	Exit^c^
Parameter	Hazard ratio (95% CI)	Hazard ratio (95% CI)	Hazard ratio (95% CI)
Number of individual measurement points (*n*/*obs*.)	1,057/3,571	1,143/3,982	1,217/4,749
Number of transitions (*n*/*obs*.)	296/339	594/754	334/396
Model 1			
Reasons for working (1–5)			
Financial	1.10 (1.01, 1.20)*	0.95 (0.90, 0.99)*	0.96 (0.89, 1.04)
Social	1.05 (0.90, 1.23)	1.18 (1.07, 1.30)**	1.14 (1.00, 1.31)*
Personal	1.07 (0.92, 1.25)	0.71 (0.65, 0.78)***	0.67 (0.59, 0.76)***
Organizational	0.97 (0.90, 1.04)	1.04 (0.99, 1.09)	1.09 (1.02, 1.16)**
−2 × Log-likelihood	3,291.29	4,240.02	3,291.29
Model 2			
Reasons for working (1–5)			
Financial	1.09 (0.96, 1.25)	0.91 (0.84, 0.99)*	0.86 (0.77, 0.97)*
Social	0.99 (0.80, 1.23)	1.14 (1.00, 1.29)*	1.29 (1.07, 1.56)**
Personal	1.24 (0.99, 1.54)	0.71 (0.63, 0.81)**	0.59 (0.50, 0.70)***
Organizational	1.01 (0.91, 1.12)	1.01 (0.95, 1.08)	1.09 (1.00, 1.19)*
Years in retirement	0.95 (0.86, 1.04)	1.00 (0.94, 1.06)	1.15 (1.06, 1.24)**
Retirement age	0.92 (0.83, 1.02)	1.01 (0.95, 1.08)	0.99 (0.91, 1.09)
Gender (male = 0, female = 1)	0.73 (0.53, 1.01)	1.35 (1.09, 1.67)*	1.50 (1.13, 1.99)**
Partner (no = 0, yes = 1)	2.33 (1.17, 4.65)*	0.78 (0.57, 1.05)	0.81 (0.51, 1.30)
Partner working (no = 0, yes = 1)	1.23 (0.91, 1.67)	0.80 (0.66, 0.97)*	0.71 (0.54, 0.93)*
Pension income (transformed)	1.00 (0.95, 1.06)	1.04 (0.98, 1.10)	0.97 (0.91, 1.04)
Involuntary retirement	1.20 (1.03, 1.39)*	1.06 (0.94, 1.19)	1.17 (1.02, 1.35)*
Occupational status (white-collar = 0, blue-collar = 1)	0.85 (0.63, 1.16)	1.32 (1.07, 1.61)*	0.81 (0.60, 1.09)
Caregiving (no = 0, yes = 1)	0.96 (0.71, 1.29)	0.92 (0.75, 1.11)	0.87 (0.66, 1.13)
Depressive symptoms	1.02 (0.96, 1.08)	1.02 (0.99, 1.05)	1.04 (0.99, 1.08)
Functional limitations	0.72 (0.53, 0.98)*	1.02 (0.85, 1.23)	0.92 (0.71, 1.20)
−2 × Log-likelihood	1,852.20	2,432.46	4,459.64

*Notes*: The covariate effects are constrained to be equal across all types of increase, decrease, and exit transitions. CI = confidence interval.

^a^Increase in work extent.

^b^Decrease in work extent.

^c^Exit from the labor market.

*p < .05, **p < .01, ***p < .001.

The middle column displays the results for decrease in work engagement. Retirees were less likely to reduce work hours if they reported financial and personal motives (supporting H1b and H2b), while those working for social reasons were more likely to decrease work (supporting H3a). No effect was found for organizational motives. Women and blue-collar workers were more likely to reduce work, while retirees with a working spouse were less likely to decrease their work extent. No effects were found for retirement age, time spent in retirement, relationship status, pension income, involuntary retirement, caregiving, depressive symptoms, or functional limitations.

The right column shows the associations for exit from work. Retirees working for personal reasons were less likely to stop working (supporting H3b), while social and organizational motives increased the likelihood to exit work (supporting H2c and H4b). The financial motive was unrelated to exit in Model 1, but decreased the likelihood to stop working in Model 2 (when demographic factors were accounted for; supporting H1c). Women, involuntary retirees, and retirees who had spent more time in retirement were more likely to exit work, and retirees with a working spouse were less likely to stop working. Retirement age, relationship status, pension income, occupational status, caregiving, depressive symptoms, and functional limitations were unrelated to exit from work.

## Discussion

While a growing body of literature highlights the diversity of pathways to retirement as well as the characteristics of those who engage in postretirement work, much less is known about the temporal dynamics of this process. In this study, we examined the extent to which retirees increase, decrease, and exit their work engagement in retirement. The results showed substantial variability in the level of engagement, but also in how it changes over time. Although the majority gradually reduced their work hours in retirement, a substantial proportion of the retirees increased their level of engagement over time, challenging the assumption of a one-directional gradual shift toward complete labor force withdrawal. We further investigated if the motives for taking up postretirement work account for individual variability in this process. Our findings suggest that financial, social, personal, and organizational (demand-driven) motives differ in how they relate to changes in work participation following retirement.

The present article adds to previous knowledge on postretirement employment by showing that work-related motivational drivers predict not only the preference for (Hess et al., 2021; van Solinge et al., 2021) and participation in (Fasbender et al., 2016; Zhan et al., 2015) postretirement work, but also the extent to which retirees change their level of engagement over time. The finding that different motives have differential implications for changes in work extent improve our understanding of postretirement work in several important ways. First, an increase in work engagement in retirement was more likely among those who work for financial reasons, but this association was not shown when accounting for factors that may restrict agency in the opportunity for postretirement work (Carr et al., 2021). This finding might imply that among these workers part-time work does not necessarily constitute the preferred level of work engagement, and that retirees with poor pension income have restricted agency in the number of working hours. This fits well with research carried out in the United States suggesting that one in five older part-time workers would prefer to work more hours and can be categorized as involuntary part-time workers (Silver et al., 2019). The decrease in effect size when accounting for the degree of voluntariness over the retirement transition further suggests that the financial motive may be more relevant for those who retired involuntarily.

Second, retirees working for financial and personal reasons were shown to be less likely to reduce their work engagement, while those working for social reasons were more likely to decrease and exit work. The findings align with the idea that retirees gradually seek to replace social engagements at work with social activities outside of work and that the social function of work might gradually reduce in importance over time. Functions related to the financial and personal meaning of work, on the other hand, are more difficult to find and replace elsewhere, suggesting that retirees who value these functions of work opt for continuity and therefore seek to maintain their level of work engagement over time. The fact that 73% of the retirees reported personal reasons for continuing working, and that this motive showed the largest effect sizes, additionally highlights the need to maintain the work role in retirement and that it constitutes an important driver for continuing working.

Third, while retirees working for personal reasons were more likely to continue working, we found motivations related to organizational demands to increase the likelihood to stop working. This finding implies that while organizational demand might be an important driver for older workers to continue their work beyond retirement age, it is insufficient for maintaining retirees in the labor force in the long run. Employers interested in retaining working retirees should therefore pay attention to the diversity of reasons for taking up work in retirement. Especially employers who are concerned about competence shortage due to generational shifts in organizations should seek to accommodate the needs of the employee to facilitate personal, social, and financial motivations for continuing working.

Taken together, the findings show that motives matter for understanding the temporal dynamics of postretirement work. By analyzing annual changes in work extent over a period of 6 years in a population-based sample of Swedish older adults, we were able to identify individual differences in how retirees change their work engagement in retirement. However, it should be noted that not all our hypotheses were supported, and more research on the role of social and organizational drivers for change in postretirement work extent is needed. To the best of our knowledge, this is the first study to investigate the temporal dynamics of postretirement work. The fact that we were able to differentiate the level of work engagement in retirement is a strength and novel contribution to the literature. However, several limitations should be noted. First, this study has been carried out in Sweden, a country with a strong welfare system and a well-developed employment protection legislation for older workers (Hyde & Dingemans, 2017). The flexibility of the pension system combined with a relatively low replacement rate (OECD, 2017) may explain why a substantial proportion of the retirees engage in postretirement work. At the same time, only a minority of the retirees reported financial motives. As the individual agency in decisions regarding postretirement work engagement may be constrained by structural factors (Damman & Henkens, 2017), it is important to examine the nature of these associations across countries with different contextual settings. Financial motives may, for example, be more prevalent in countries with a less developed social security system (Hershey et al., 2010), but it is still unknown if such differences also influence the association with work participation.

Second, our measures of motives for postretirement work were based on single items, which may have reduced content validity. Future studies need to include more refined measures on motivations for postretirement work. Especially the nature of organizational demand, the motivational drivers in response to those demands, as well as the extent to which they relate to changes in postretirement work engagement are important outlets for future research. Motives related to the social function of work may also vary depending on the need for other forms of social engagements, such as volunteer work or caregiving (Gonzales & Nowell, 2017; Gonzales et al., 2017). A detailed understanding of different types of motivational drivers and how they shape work and retirement pathways, therefore, requires attention to the influence of household, organizational, and institutional factors (National Academies of Sciences, Engineering, and Medicine, 2022). Finally, it should be noted that some of the data collection (waves 6 and 7) was conducted during the coronavirus disease 2019 pandemic. Although Sweden remained relatively open during this period (with few mandatory restrictions) it is possible that both motives and opportunities to work were affected, and the implications for changes in postretirement work engagement remains an important area for future research.

Another important outlet for future research is to examine if different motives are more or less prevalent depending on time spent in retirement, and if motives vary in importance for change in postretirement work participation depending on retirement duration, employment change, or if the retiree returns to work after an initial labor force withdrawal. For instance, our findings suggest that the social function of work gradually decreases in importance, but we did not investigate if this motive is more common in the first years following retirement. More knowledge about organizational demands in relation to the retirement event could also contribute to a better understanding of how employers can motivate older workers to continue working.

## Conclusions

The present study contributes to previous knowledge on postretirement employment by highlighting the diversity of work engagement in retirement, but also the extent to which it changes over time. Our findings suggest that financial motives constitute an important driver for taking up more work in retirement, while motives related to the personal meaning of work explain why retirees maintain their level of engagement over time. The social function of work, on the other hand, might decrease in importance for maintaining work-related social ties, resulting in a gradual disengagement from the labor force. The fact that organizational motives increased the likelihood to stop working further highlights the need to acknowledge the diversity of motives for continuing working, and that employer demand without the presence of internal motivations may be insufficient to retain older workers in the labor force.

## Supplementary Material

gbac130_suppl_Supplementary_Tables
