# Supplementary material for: Motivational Drivers of Temporal Dynamics in Postretirement Work
Source: J Gerontol B Psychol Sci Soc Sci. 2022 Sep 8;78(1):179–89. doi: 10.1093/geronb/gbac130 (PMC9890924; doi:10.1093/geronb/gbac130)
Supplement: gbac130_suppl_Supplementary_Tables [file gbac130_suppl_supplementary_tables.pdf]

**Motivational Drivers of Temporal Dynamics in Post-Retirement Work**

**Supplementary Material**

**Table S1**

*Bivariate Correlations of Reasons for Working within and across Measurement Waves.*

| | | Financial (F) | | | | | Social (S) | | | | | Personal (P) | | | | | Organizational (O) | | | | |
|---|---|---|---|---|---|---|---|---|---|---|---|---|---|---|---|---|---|---|---|---|---|
| | *n* | T2 | T3 | T4 | T5 | T6 | T2 | T3 | T4 | T5 | T6 | T2 | T3 | T4 | T5 | T6 | T2 | T3 | T4 | T5 | T6 |
| F T2 | 450 | | | | | | | | | | | | | | | | | | | | |
| F T3 | 568 | .75 | | | | | | | | | | | | | | | | | | | |
| F T4 | 603 | .66 | .72 | | | | | | | | | | | | | | | | | | |
| F T5 | 662 | .68 | .71 | .78 | | | | | | | | | | | | | | | | | |
| F T6 | 670 | .50 | .67 | .74 | .77 | | | | | | | | | | | | | | | | |
| S T2 | 456 | -.01 | -.06 | -.05 | -.01 | .01 | | | | | | | | | | | | | | | |
| S T3 | 587 | -.03 | .04 | .01 | -.05 | -.04 | .55 | | | | | | | | | | | | | | |
| S T4 | 623 | .003 | .04 | .05 | .03 | -.03 | .51 | .62 | | | | | | | | | | | | | |
| S T5 | 678 | .02 | .05 | .04 | .02 | .01 | .53 | .59 | .63 | | | | | | | | | | | | |
| S T6 | 685 | -.01 | .05 | .08 | .07 | .07 | .49 | .60 | .57 | .65 | | | | | | | | | | | |
| P T2 | 460 | -.03 | -.13 | -.23 | -.20 | -.01 | .51 | .28 | .26 | .22 | .29 | | | | | | | | | | |
| P T3 | 588 | .004 | .01 | .01 | -.12 | -.01 | .34 | .61 | .37 | .38 | .35 | .52 | | | | | | | | | |
| P T4 | 616 | -.02 | -.02 | .002 | -.05 | -.03 | .25 | .43 | .55 | .39 | .33 | .54 | .61 | | | | | | | | |
| P T5 | 675 | -.02 | -.04 | -.06 | -.13 | -.13 | .27 | .38 | .32 | .52 | .31 | .52 | .58 | .61 | | | | | | | |
| P T6 | 682 | -.10 | -.06 | -.06 | -.08 | .001 | .25 | .41 | .33 | .36 | .54 | .52 | .58 | .53 | .59 | | | | | | |
| O T2 | 444 | -.05 | -.07 | -.18 | -.15 | -.15 | .12 | .16 | .02 | .07 | .05 | .04 | .10 | .06 | -.01 | -.03 | | | | | |
| O T3 | 564 | -.03 | .02 | -.01 | -.04 | .01 | .15 | .21 | .12 | .15 | .11 | .04 | .15 | .10 | .12 | .12 | .56 | | | | |
| O T4 | 604 | .03 | .03 | -.04 | -.08 | -.04 | .06 | .16 | .11 | .07 | .07 | -.11 | .05 | .07 | -.02 | -.03 | .60 | .70 | | | |
| O T5 | 655 | -.02 | .06 | -.01 | .01 | -.01 | .05 | .17 | .02 | .15 | .10 | -.13 | .03 | -.07 | -.02 | .01 | .52 | .61 | .69 | | |
| O T6 | 657 | -.10 | .005 | -.01 | .08 | .02 | .07 | .12 | .03 | .12 | .16 | .11 | .01 | -.08 | .01 | .11 | .38 | .43 | .55 | .65 | |
| *M* | | 2.17 | 2.20 | 2.31 | 2.25 | 2.12 | 3.83 | 3.83 | 3.87 | 3.86 | 3.89 | 3.92 | 3.94 | 3.91 | 3.99 | 4.06 | 2.79 | 2.87 | 2.98 | 2.97 | 2.93 |
| *SD* | | 1.43 | 1.39 | 1.45 | 1.45 | 1.37 | 1.04 | 1.07 | 1.06 | 1.04 | 1.06 | 1.05 | 1.09 | 1.09 | 1.03 | 1.02 | 1.67 | 1.71 | 1.69 | 1.67 | 1.68 |

*Note*. Measure not available in T1 and T7.

**Table S2**

*Mean, Standard Deviations, and Correlations of the Study Variables.*

| | | *M/%* | SD | 1. | 2. | 3. | 4. | 5. | 6. | 7. | 8. | 9. | 10. | 11. | 12. | 13. | 14. | 15. |
|---|---|---|---|---|---|---|---|---|---|---|---|---|---|---|---|---|---|---|
| 1. | Post-retirement work (0–4) | 0.75 | 1.24 | | | | | | | | | | | | | | | |
| 2. | Financial motive[a] (1–5) | 2.21 | 1.42 | .14 | | | | | | | | | | | | | | |
| 3. | Social motive[a] (1–5) | 3.86 | 1.05 | .08 | .03 | | | | | | | | | | | | | |
| 4. | Personal motive[a] (1–5) | 3.97 | 1.06 | .21 | -.03 | .61 | | | | | | | | | | | | |
| 5. | Organizational motive[a] (1–5) | 2.91 | 1.68 | -.03 | -.03 | .17 | .08 | | | | | | | | | | | |
| 6. | Years in retirement[a] (0–11) | 3.12 | 2.07 | -.18 | -.02 | -.01 | .02 | -.06 | | | | | | | | | | |
| 7. | Retirement age[b] (61–71) | 64.16 | 1.50 | .02 | -.06 | .10 | .10 | .16 | -.29 | | | | | | | | | |
| 8. | Gender[b] (% female) | 54.00 | | -.15 | .12 | .13 | .07 | .13 | .02 | 0.01 | | | | | | | | |
| 9. | Relationship status[a] (% with partner) | 77.39 | | -.01 | -.18 | -.05 | -.02 | .03 | .03 | -0.09 | -.08 | | | | | | | |
| 10. | Spouse's work status[a] (% working) | 28.15 | | .31 | .08 | -.01 | .05 | -.05 | -.23 | -0.03 | -.23 | -.03 | | | | | | |
| 11. | Pension income[b] (transformed[c]) | 2.75 | 1.79 | -.08 | -.26 | <.01 | .03 | -.06 | <.01 | 0.11 | -.25 | .05 | -.03 | | | | | |
| 12. | Involuntary retirement[b] (1–5) | 1.34 | 0.92 | <.01 | .16 | .06 | .08 | -.06 | -.04 | 0.12 | .01 | -.09 | <.01 | .01 | | | | |
| 13. | Occupation (% blue-collar) | 32.89 | | <.01 | .15 | .01 | -.02 | -.14 | .03 | -0.07 | -.07 | -.01 | .02 | -.19 | <.01 | | | |
| 14. | Caregiving[a] (% yes) | 34.78 | | -.01 | -.05 | .03 | .04 | .01 | -.02 | 0.00 | .08 | .09 | -.03 | .04 | -.03 | <.01 | | |
| 15. | Depressive symptoms[a] (0–33) | 3.49 | 3.66 | -0.03 | .21 | -.05 | -.06 | <.01 | .02 | 0.08 | .05 | -.16 | .01 | -.06 | .17 | <.01 | -.03 | |
| 16. | Functional limitations[a] (0–2) | 0.41 | 0.55 | -0.01 | .15 | -.08 | -.07 | <.01 | .04 | 0.05 | <.01 | -.05 | .02 | -.05 | .14 | <.04 | -.01 | .34 |

*Note*. [a]Time variant. [b]Time invariant. [c]1 = 100,000 SEK.
